# Integration of Human Protein Sequence and Protein-Protein Interaction Data by Graph Autoencoder to Identify Novel Protein-Abnormal Phenotype Associations

**DOI:** 10.3390/cells11162485

**Published:** 2022-08-10

**Authors:** Yuan Liu, Ruirui He, Yingjie Qu, Yuan Zhu, Dianke Li, Xinping Ling, Simin Xia, Zhenqiu Li, Dong Li

**Affiliations:** 1 State Key Laboratory of Proteomics, Beijing Proteome Research Center, National Center for Protein Sciences (Beijing), Beijing Institute of Lifeomics, Beijing 102206, China; 2 College of Life Sciences, Hebei University, Baoding 071002, China; 3 School of Basic Medical Sciences, Anhui Medical University, Hefei 230032, China

**Keywords:** deep learning, graph autoencoder, protein-phenotype associations prediction

## Abstract

Understanding gene functions and their associated abnormal phenotypes is crucial in the prevention, diagnosis and treatment against diseases. The Human Phenotype Ontology (HPO) is a standardized vocabulary for describing the phenotype abnormalities associated with human diseases. However, the current HPO annotations are far from completion, and only a small fraction of human protein-coding genes has HPO annotations. Thus, it is necessary to predict protein-phenotype associations using computational methods. Protein sequences can indicate the structure and function of the proteins, and interacting proteins are more likely to have same function. It is promising to integrate these features for predicting HPO annotations of human protein. We developed GraphPheno, a semi-supervised method based on graph autoencoders, which does not require feature engineering to capture deep features from protein sequences, while also taking into account the topological properties in the protein–protein interaction network to predict the relationships between human genes/proteins and abnormal phenotypes. Cross validation and independent dataset tests show that GraphPheno has satisfactory prediction performance. The algorithm is further confirmed on automatic HPO annotation for no-knowledge proteins under the benchmark of the second Critical Assessment of Functional Annotation, 2013–2014 (CAFA2), where GraphPheno surpasses most existing methods. Further bioinformatics analysis shows that predicted certain phenotype-associated genes using GraphPheno share similar biological properties with known ones. In a case study on the phenotype of abnormality of mitochondrial respiratory chain, top prioritized genes are validated by recent papers. We believe that GraphPheno will help to reveal more associations between genes and phenotypes, and contribute to the discovery of drug targets.

## 1. Introduction

Phenotypes refer to observable physical or biological traits of an organism. Revealing the relationships between genes/proteins and their related phenotypes is critical for designing diagnosis, therapy and prevention strategies against diseases [1]. Human Phenotype Ontology (HPO) is a standardized vocabulary for describing the phenotypic abnormalities associated with human diseases [2]. At present, only small quantities of human protein-coding genes (~3500) have HPO annotations, and a large number of phenotype-associated genes/proteins remain undiscovered. It is laborious to experimentally identify the associations between proteins and abnormal phenotypes. Therefore, a robust computational strategy is desirable to systematically identify the potential phenotype-associated genes/proteins at the proteome scale.

HPO annotation prediction is essentially a large-scale multi-label classification problem, which is also well-known as automated function prediction (AFP). To advance the performance of AFP, several Critical Assessment of Functional Annotation (CAFA) challenges [3,4,5] were held in recent years, and many computational strategies have been proposed to address the problem of protein function prediction. The Critical Assessment of Functional Annotation challenges (CAFA) organizers first provided a large number of proteins for researchers to develop their algorithms for associating these proteins with Gene Ontology terms or Human Phenoytpe Ontology terms. Additionally, organizers then spent several months verifying these proteins’ functions by experiments. Those verified proteins’ functional annotation constituted the CAFA benchmark data, to assess these computational methods [3,4,5]. The existing computational approaches for AFP are mostly sequence-based or protein–protein interaction network (PPI network)-based methods.

For sequence feature, the GoFDR (Gene Ontology—functionally discriminating residues) method uses BLAST to find homologous sequences and transfers their functional annotations to the query protein [6]. Funfam (Functional families) method uses domains and motifs to indicate the function of a protein [7]. For PPI network feature, several semi-supervised methods such as guilt by association [8], random walk [9], and weighted score computation [10] are used to capture the topological information in the PPI network. The GeneMANIA prediction server [10] integrates multiple networks in a linear regression fashion to learn the weights over multiple networks. Given a single query gene, GeneMANIA finds genes likely to share function with the query gene based on their interactions with it. Recently, HPODNets presents a deep GCN architecture to capture high-order topological information from multiple protein–protein interaction networks [11].

Integrating sequence and PPI network information would be promising to improve the prediction performance of AFP. In fact, several approaches (MS-kNN [12], HPOLabeler [13], GOLabeler [14], Phenostruct [15], DeepGO [16] and DeepGOPlus [17]) of using the idea of integration have been already proposed. MS-kNN (Multi-Source k-Nearest Neighbor) algorithm predicts the functions by averaging over the prediction scores from three data sources [12]. HPOLabeler [13] and GO-Labeler [14] use Learning to Rank to integrate multiple classifiers trained from different sequence-derived data. PHENOstruct [15] uses Support Vector Machine to give predictions based on multiple feature vectors. DeepGO/DeepGOPlus [16,17] encodes the PPI network and sequence feature separately then integrates them into a Convolutional Neural Network model using simple concatenation.

However, all above integration methods are rather simple feature concatenation between sequence and PPI network. Ignorance of the interaction between these features might underestimate the performance of prediction. To address this problem, we developed GraphPheno, a variational graph autoencoders based architecture, which can extend the feature extracted from protein sequences using topological information of PPI network to predict the relationships between human genes/proteins and abnormal phenotypes. GraphPheno does not rely on any manually crafted features and is entirely data driven. Cross validation and independent dataset test were performed to test the prediction ability of GraphPheno. Then, the performance of our model was compared with existing classifiers, and results prove that our model has equivalent performance with other state-of-the-art classifiers. Further bioinformatics analysis shows that predicted and known phenotype-associated genes share similar biological properties. Finally, we performed a case study on HP:0008972 (Decreased activity of mitochondrial respiratory chain, DAMRC, https://hpo.jax.org/app/browse/term/HP:0008972, accessed on 18 May 2021), and the results show that our algorithm is able to identify novel relationships between human genes/proteins and abnormal phenotypes, and these newly identified DAMRC-associated genes might be potential biomarkers of multiple mitochondrial related diseases.

## 2. Materials and Methods

### 2.1. Gold Standard Data Sets

Two releases of HPO database were used. First, we downloaded the Human Phenotype Ontology (HPO) from https://hpo.jax.org (accessed on 3 June 2019). We expanded every gene-phenotype association in the original dataset to all the ancestor terms of the phenotype so that we could fully utilize existing data for gene-phenotype association prediction. This expansion was done using true path rule [18], that is, if a gene is annotated with a HPO term *T*, we consider that this gene is also inherently annotated with all the ancestors of *T*. We divided all the HPO terms into six groups, according to the number of appearances (in brackets): Very rare (1–3), Rare (4–10), Uncommon (11–30), Common (31–100), Very common (100–300) and Extremely common (>300). Supplementary Figure S1 shows the statistics of these six groups. These statistics indicate that HPO terms with low appearances, i.e., Very rare, Rare, occupy 37.68% and 20.74%, respectively, of all HPO terms. Whereas these HPO terms are only small parts of all protein–HPO term pairs, i.e., Very rare and Rare, occupying only 1.04%, 2.20%, respectively, of all pairs. To more confidently assess the performance in predicting individual terms, terms with no more than 10 annotated proteins were removed [4]. We obtained 496,202 gene-phenotype associations with an average of 126 HPO annotations per gene (Supplementary Table S1). Then, we downloaded HPO (April 2021) and constricted a novel independent test set of 128,115 gene-phenotype associations that were newly included in the database between June 2019 and April 2021 (none of them was used in the cross-validation for training). Since there is no existing experimentally negative data set, we built our gold standard negative data sets based on unannotated genes for each HPO term. For each HPO term, we randomly selected the same number of genes as the annotated genes from the non-annotated genes of the phenotype to construct the gold standard negative data set. The gold standard negative data set contains 496,202 gene-phenotype associations for cross validation and 128,115 associations for independent test as well.

### 2.2. Comparison Dataset

To compare our method with other phenotype prediction methods, we generated a comparison dataset on automatic HPO annotation for no-knowledge proteins following the CAFA2 challenge rules [4]. The training dataset contains phenotype annotations that were available before January 2014. Additionally, the testing dataset (benchmark) of the CAFA2 challenge was collected from annotations that appeared during January 2014 until September 2014 (Supplementary Table S2). Proteins in the benchmark of the CAFA2 challenge are not associated with any HPO terms before the submission date (January 2014), and received HPO annotations by September 2014.

### 2.3. Protein–Protein Interaction Network and Sequence Evidence for Prediction

Protein sequences were downloaded from UniProt [19] PPI network was downloaded from the STRING database (v11.0) [20].

Amino acid sequences were encoded following the conjoint triad (CT) method [21], which has been widely used to represent sequences in related fields. 20 kinds of amino acids are first clustered into 7 classes according to the dipoles and volumes of the side chains. Then, all the amino acids in the same class are considered identical. The mapping between classes and amino acids is shown in Supplementary Table S3. The conjoint triad considers the properties of one amino acid and its vicinal amino acids, and regards any three continuous amino acids as a unit, then, the triad frequencies were counted by calculating the occurrence numbers within the protein sequence. Thus, the dimension of the CT encoding feature vector is 7 × 7 × 7 = 343. Using the CT method, we manage to convert amino acid sequences into fixed-dimension representation.

For the PPI network data downloaded from STRING, we use the “combined score” provided by STRING as the confidence score. Interactions with a “combined score” greater than 300 were used to construct PPI network [22]. Then, PPI network was converted to the format of adjacency matrix.

### 2.4. Variational Graph Autoencoder Model

Variational graph autoencoder (VGAE) [23] is an unsupervised feature extraction method, which can generate latent representations based on both network structure and node features by training the encoder and decoder at the same time.

We consider the PPI network as an undirected graph, *G* = (*V*,*E*,*X*), where {*v_i_*}_*i* = 1,…,*n*_ consists of a set of proteins in the graph and *e*_*i*,*j*_ = <*v_i_*,*v_j_*>∈*E* represents one interaction in the PPI network. The topological structure of graph *G* was represented by an adjacency matrix *A*, and we incorporated the combined score as weights: *A*_*i*,*j*_ = combined score/1000 if *e*_*i*,*j*_∈*E*, otherwise *A*_*i*,*j*_ = 0 (we assume that every node is connected to itself and set diagonal elements to 1). *x_i_*∈*X* indicates the content features associated with each node *v_i_*, which is the encoding of protein sequence using the CT method. The graph encoder is constructed using graph convolutional network (GCN). The goal of encoder is to map the original features *X* to the latent variable *Z* with the network information *A*. Z is a numerical matrix of protein-embedding vectors, which is also interpretable latent representation for undirected graphs learnt using VGAE. Z was used to compress the complex graph structure data in non-Euclidean space into simple low-dimensional numerical vectors while considering the relevant information of the original input as completely as possible.

We define a spectral convolution function *f_gcn_* [23]:

(1) $$Z^{\left(l+1\right)}=f_{gcn}(Z^{\left(l\right)},A|W^{\left(l\right)})$$

Here, *Z*^(*l*)^ is the input for convolution, and *Z*^(*l*+1)^ is the output after convolution. We have *Z*^0^ = *X* in our work. *W*^(l)^ is a matrix of filter parameters we need to learn in the neural network. Each layer of our graph convolutional network can be defined as follows:

(2) $$f_{gcn}(Z^{\left(l\right)},A|W^{\left(l\right)})=\Phi \left(\tilde{A}Z^{\left(l\right)}W^{\left(l\right)}\right)$$

where *A* = *D*^−½^ (*A* + *I*)*D*^−½^, i.e., the symmetrically normalized adjacency matrix *D_ij_* = ∑*_j_A_ij_*. *I* is the identity matrix of *A* and *Φ* is activation function *leakyRelu* (*t*) = *max*(0, *t*) [24].

Our graph encoder consists of two GCN layers, and we let the prior over the latent variables Z be the centered isotropic multivariate Gaussian [25]:

(3) $$q(Z|X,A)=\overset{n}{\underset{i=1}{\prod }}q(z_{i}|X,A)$$

(4) $$q(z_{i}|X,A)=N(z_{i}|\mu_{i},diag(\sigma^{2}))$$

where *μ* and *σ* are the mean and variance, respectively, of the Gaussian distribution corresponding to latent variable *Z*:

(5) $$z_{i}\;=\mu_{i}+\sigma_{i}⊙\epsilon_{i}$$

where ⊙ is element-wise multiplication and *ϵ_i_*~*N* (0,1).

Next, we define a simple inner product decoder that aims to reconstruct adjacency matrix *A* using learned latent variable *Z*:

(6) $$p(\tilde{A}|Z)=\overset{n}{\underset{i=1}{\prod }}\overset{n}{\underset{j=1}{\prod }}p({\tilde{A}}_{ij}|z_{i}z_{j})$$

(7) $$p({\tilde{A}}_{ij}=1|z_{i}z_{j})=sigmoid\left(z_{i},z_{j}{}^{T}\right)$$

Finally, to make the approximated adjacency matrix $\tilde{A}$ and the original adjacency matrix *A* as close as possible, we optimize the model using the following loss function:

(8) $$L=E_{q(Z|\left(X,A\right))}\left[log\;p(\tilde{A}|Z)\right]-KL\left[q(Z|X,A)∥p\left(Z\right)\right]$$

where *KL*[*q* (∙)∥*p* (∙)] is the Kullback–Leibler divergence [26] between *q* (∙) and *p* (∙). Here, we assume *p* (*Z*)~*N* (0,1). The cost function reflects how accurately our model can reconstruct the input network and how closely the latent variables can match *p* (*Z*). Stochastic gradient descent was used to train the model in order to optimize the cost function with respect to the parameters of the encoder.

### 2.5. Neural Network Model

NN has become one of the most popular and powerful techniques for supervised learning [27]. We take *Z* as final protein feature vectors and train a two-layer NN as the final supervised classifier. We define a function *f_mlp_*:

(9) $$f_{mlp}(P^{\left(l\right)}|W^{\left(l\right)},P^{\left(l\right)})=leakyReLU\left(W^{\left(l\right)}P^{\left(l\right)}+b^{\left(l\right)}\right)$$

Each layer of our NN can be defined as follows:

(10) $$P^{\left(l+1\right)}=f_{mlp}(P^{\left(l\right)}|W^{\left(l\right)},b^{\left(l\right)})$$

Here, *P*^(*l*)^ is the input for NN, and *P*^(*l*+1)^ is the output after each NN layer. We have *P*^0^ = *Z* in our work. *W*^(*l*)^ is a matrix of filter parameters we need to learn in the neural network, *b*^(*l*)^ is the bias of each layer.

Our NN consists of two layers, and we add batch normalization and dropout between each layer:

(11) $$P^{\left(1\right)}=f_{mlp}(Z|W^{\left(0\right)},b^{\left(0\right)})$$

(12) $$P^{\left(2\right)}=f_{mlp}(P^{\left(1\right)},|W^{\left(1\right)},b^{\left(1\right)})$$

We compute the final probability using sigmoid function:

(13) $$P^{\left(3\right)}=sigmoid\left(P^{\left(2\right)}\right)$$

For the NN classifier, it is a multi-class, multi-label model, and the dimension of the output space is the number of HPO terms within each ontology. Each protein may be predicted with multiple HPO terms simultaneously. The classifier predicts the probabilities of the protein having each HPO term annotation.

### 2.6. Cross-Validation

To test the efficacy of the overall performance of various assessment models, the five-fold cross-validation protocol was used. The gold standard positive and negative data sets were randomly divided into five approximately equal subsets. Four subsets were used as training data sets to train the model, and the remaining one was used as the test data set to calculate the AUC.

### 2.7. F-Max for Protein-Centric Evaluation

Protein-centric evaluation measures how accurately methods can assign functional terms to a protein. We calculated the *F*-measure (a harmonic mean between precision and recall) for each threshold and obtained its maximum value over all thresholds. Precision (*pr*), recall (*rc*), and the resulting *F*-max are defined as:

(14) $$pr\left(t\right)=\frac{1}{m\left(t\right)}\overset{m\left(t\right)}{\underset{i=1}{\sum }}\frac{\sum_{f}I\left(f\in P_{i}\left(t\right)\Lambda f\in T_{i}\right)}{\sum_{f}I\left(f\in P_{i}\left(t\right)\right)}$$

(15) $$rc\left(t\right)=\frac{1}{n}\sum_{i=1}^{n}\frac{\sum_{f}I\left(f\in P_{i}\left(t\right)\Lambda f\in T_{i}\right)}{\sum_{f}I\left(f\in T_{i}\right)}$$

(16) $$F-max=\underset{t}{\max}\left\{\frac{2\cdot pr\left(t\right)\cdot rc\left(t\right)}{pr\left(t\right)+rc\left(t\right)}\right\}$$

where $P_{i}$ denotes the set of terms that have predicted scores greater than or equal to t for a protein i, $T_{i}$ denotes the corresponding ground-truth set of terms for that protein, $m\left(t\right)$ is the number of proteins with at least one predicted score greater than or equal to t, $I\left(\cdot \right)$ is an indicator function, and n is the total number of proteins.

### 2.8. AUC for Term-Centric Evaluation

Term-centric evaluation measures how accurately methods can assign proteins to a functional term. Here, we use AUC (the area under ROC curve) for term-centric evaluation. The ROC curve can show the efficacy of one test by presenting both sensitivity and specificity for different cutoff points. For cutoff t and HPO term f, sensitivity (Sn) and specificity (Sp) are defined as:

(17) $$Sn\left(t\right)=\frac{\sum_{i=1}^{n}I\left(f\in P_{i}\left(t\right)\Lambda f\in T_{i}\right)}{\sum_{i=1}^{n}I\left(f\in T_{i}\right)}$$

(18) $$Sp\left(t\right)=\frac{\sum_{i=1}^{n}I\left(f\notin P_{i}\left(t\right)\Lambda f\notin T_{i}\right)}{\sum_{i=1}^{n}I\left(f\notin T_{i}\right)}$$

We then computed ${\mathrm{AUC}}_{f}$ of term f using the ROC curve obtained by plotting (1 − *Sp*$\left(t\right)$,$Sn\left(t\right)$) changing t, and obtained the final AUC by averaging ${\mathrm{AUC}}_{f}$ over all terms. An ideal test with perfect discrimination (100% sensitivity, 100% specificity) has an AUC of 1.0, whereas a non-informative prediction has the AUC of 0.5, indicating that it may be achieved by mere guess. The more a test’s AUC approximates to 1.0, the higher its overall efficacy will be.

## 3. Results

### 3.1. Overview of Our Prediction Protocol

**Figure 1:**
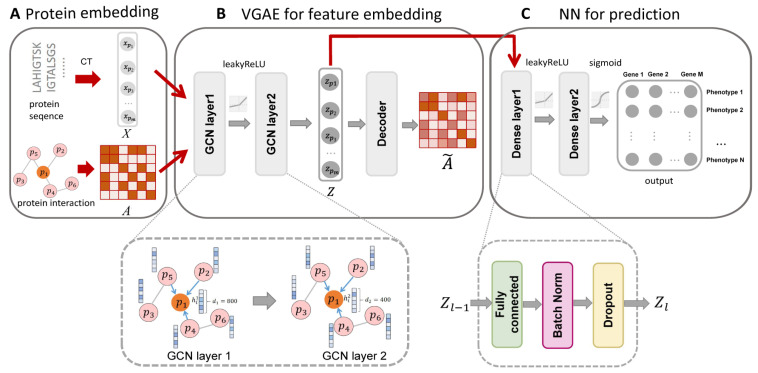
Overview of GraphPheno. The model consists of three modules: (**A**) the Protein embedding module: this module takes proteins interaction and sequence information as input. PPI network was converted to the format of adjacency matrix *A*. Proteins amino acid sequences were embedded using the conjoint triad (CT) method and served as proteins initial features *X*; (**B**) the VGAE module for feature embedding: This module consists of a two-layer GCN encoder and a dot product decoder, and generates latent representations *Z* based on both topological information from PPI network and protein sequence features. The adjacency matrix $\tilde{A}$ is reconstructed using the latent variable *Z* through the dot product decoder. (**C**) Neural network module for prediction: Gold standard dataset is used to train this module. This module takes VGAE embedding as an input to produce prediction scores for each gene-phenotype association.

We have developed a protein-phenotype associations prediction pipeline. The first stage of GraphPheno is protein embedding. The model takes STRING PPI network and proteins amino acid sequences as input. PPI network was converted to the format of adjacency matrix. Protein amino acid sequences were embedded using the conjoint triad (CT) method and served as the proteins’ initial features (Figure 1A). The second stage of GraphPheno is an unsupervised feature extraction based on variational graph autoencoder (VGAE) [23], which can generate latent representations based on both topological information from PPI network and protein sequence features (Figure 1B). The purpose of VGAE is to learn interpretable embedding for each protein by training the encoder and decoder at the same time. The encoder is a two-layer GCN architecture, which can map the original features *X* to the latent variable *Z* with the network information *A*. As our latent embedding already contains both node attributes and sequence information, we define a simple inner product decoder that aims to reconstruct adjacency matrix *A* using learned latent variables *Z*. In the third stage of GraphPheno, as shown in Figure 1, we take *Z* as the final protein feature vectors and train a two-layer NN as the final supervised classifier. Each dense layer consists of three parts: “Fully connected layer”, “Batch Normalization” and “Dropout”. The inputs to the NN are feature vectors for each protein, whereas the output layer represents HPO terms that we aim to predict (Figure 1C).

### 3.2. Performance Evaluation for GraphPheno

To verify the effectiveness of model integrating protein sequences and PPI network, we constructed six baseline methods.

Baseline 1, ‘Sequence’: Variational autoencoder was used to generate feature vectors based on protein sequences, and then NN (Neural Network) was used to give prediction scores for each protein-phenotype associations. 

Baseline 2, ‘BLAST’: Blast was used to retrieve the query protein against the gold standard dataset, and make predictions about a protein-phenotype association based on the number of blast hits with proteins annotated with the HPO term divided by the total number of blast hits.

Baseline 3, ‘PPI network’: The prediction scores for each protein-phenotype association were calculated as the number of interaction partners with the query protein which are also annotated to the query HPO term, divided by total number of query protein’s interaction partners in the STRING PPI network.

Baseline 4, ‘PPI network (VGAE)’: VGAE was used to generate feature vectors based on PPI network, and then NN was used to generate prediction scores for predicted protein-phenotype associations.

Baseline 5, ‘Concatenation’: To verify the effectiveness of using graph autoencoder for data integration, we constructed a baseline integration method called ‘Concatenation’. This method integrates protein sequences and PPI network by using a simple vector concatenation protocol.

Baseline 6, ‘Naive’: This method was developed by Wyatt et al. [28] for protein-centric evaluation. It assumes that there is a similar HPO term distribution for any protein. The prediction score for each protein-HPO term association was based on the number of appearances of the HPO term in the database.

**Figure 2:**
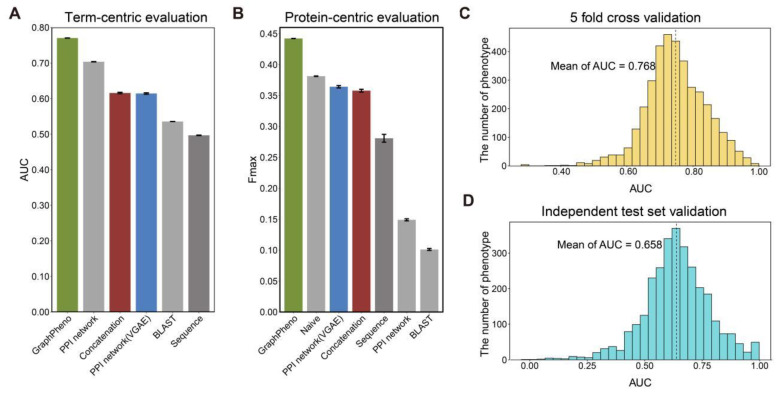
Performance evaluation of GraphPheno. (**A**,**B**) Performance comparison of various prediction models in term-centric evaluation (**A**) and protein-centric evaluation (**B**). Confidence intervals (95%) were determined using bootstrapping with 100 iterations. (**C**) The distributions of AUCs for the prediction of 3741 HPO terms by GraphPheno model against five-fold cross-validation prediction. (**D**) The distributions of AUCs for the prediction of 2993 HPO terms by GraphPheno model against independent test set validation. The mean value of AUCs are plotted in dotted lines.

The results in Figure 2A,B indicate that the GraphPheno achieved the best performance in both term-centric evaluation and protein-centric evaluation. The performance difference between GraphPheno and all other methods were all significant (*p* < 0.001) by Student’s *t* test.

Interestingly, ‘PPI network’ shows the performance second only to GraphPheno in term-centric evaluation, yet its performance decreases dramatically in protein-centric evaluation. This method makes predictions by counting the number of interaction partners with the query protein, so it is only effective when HPO term has a considerable amount of annotation genes. However, we found that HPO terms with high appearances, i.e., Very common, extremely common, occupy only small parts (7.49% and 4.32%, respectively) of all HPO terms (Supplementary Figure S1), and this could underestimate its performance in protein-centric evaluation.

In particular, ‘Naive’ shows the performance second only to GraphPheno in protein-centric evaluation. ‘Naive’ baseline uses the number of appearances of HPO term as the prediction score, but it is not possible to evaluate it for term-centric evaluation because it reports the same score for an HPO term for any gene.

In addition to the five-fold cross-validation, we assessed our model on a novel independent test set of 128,115 gene-phenotype associations that were newly included in the database between June 2019 and April 2021 (none of them was used in the cross-validation for training). The distributions of AUC for five-fold cross-validation and independent test prediction using GraphPheno of each phenotype are shown in Figure 2C,D, respectively.

**Figure 3:**
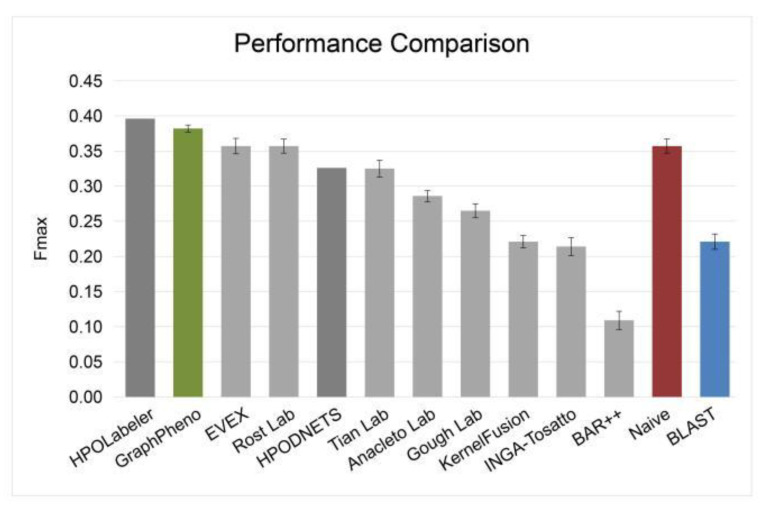
Performance comparison under the benchmark of the CAFA2 challenge using *F*-max. GraphPheno (green) was compared with the top performing CAFA2 participating methods (light gray), baseline methods in CAFA2 challenge (red for Naive, and blue for BLAST), and several HPO predicting methods proposed after CAFA2 challenge (dark gray). *F*-max is the maximum value of *F*-measure over all thresholds. Confidence intervals (95%) were determined using bootstrapping with 100 iterations.

Moreover, we conducted performance comparison on automatic HPO annotation for no-knowledge proteins under the benchmark of the CAFA2 challenge [4] as described in Method 2.2 using protein centric metric *F*-max (Figure 3). GraphPheno is compared with the top performing CAFA2 [4] participating methods (EVEX [4], Rost Lab [4], Tian Lab [4], Anacleto Lab [4], Gouph Lab [4], KernelFusion [4], INGA-Tosatto [29], BAR++ [4]), baseline methods in CAFA2 challenge (Naive, BLAST), and two HPO predicting methods proposed after CAFA2 challenge (HPOLabeler and HPODNETS). The results of eight CAFA2 participating methods and two baselines were copied from CAFA2 result announcement, and the results of HPODNETS were copied from their papers. We ran GraphPheno and HPOLabeler using the data sources released before the beginning of CAFA2 challenge (January 2014) to avoid information leakage. We found that GraphPheno outperforms all top performing CAFA2 participating methods and HPODNETS (Figure 3). In particular, HPOLabeler has slightly higher *F*-max than GraphPheno (0.396 vs. 0.383, *p*-value = 1.92 × 10^−4^, U-test). It should be noted that HPOLabeler can make use of wide variety of biological evidences from Gene Ontology [30], InterPro [31], and HPO term frequency [1]. In constract, GraphPheno only uses protein primary sequence and protein interaction data as input. For those proteins without rich annotations, GraphPheno can be a good complement to HPOLabeler.

### 3.3. Predicted and Known Phenotype-Associated Genes Share Similar Biological Properties

**Figure 4:**
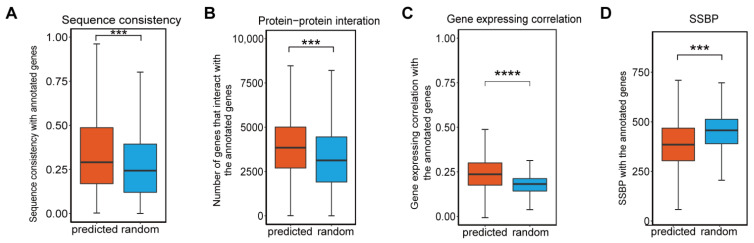
Predicted and known annotated genes of 4369 phenotypes share similar biological properties. Box plots of the sequence consistency (**A**), number of protein–protein interactions (**B**), gene expression correlation coefficient (**C**), number of proteins in the smallest shared biological process (**D**) between predicted genes and random genes with known annotated genes for each phenotype. The random genes were randomly selected from unannotated genes of each phenotype with an equal number of predicted genes. (In the box plots, the middle bar represents the median, and the box represents the interquartile range; bars extend to 1.5× the interquartile range. *p*-values are calculated by the Student’s *t*-test and shown on the top of the boxes. *** *p*-value < 0.001, **** *p*-value < 0.0001).

Next, we trained GraphPheno by all the available gene-phenotype relationships in HPO database before April 2021, and implemented a proteome-wide gene-phenotype association scanning to generate a predicted gene-phenotype association data set. We use Youden Index [32] to find cut-point with optimal threshold in five-fold cross-validation to determine gene-phenotype association authenticity. Ultimately, we predicted 21,114,059 pairs of candidate gene-phenotype associations between 18,155 genes and 4369 phenotypes. To further clarify the reliability of the model’s prediction results, we have conducted multiple bioinformatics analysis. We checked the sequence consistency and protein–protein interaction number between known annotated genes and predicted genes across 4369 phenotypes, and those between known annotated genes and random genes as controls. The random genes were randomly selected from unannotated genes of each phenotype with an equal number of predicted genes. As expected, predicted genes have a significantly higher sequence consistency and probability of interaction with known annotated genes than random genes (Figure 4A,B), which is obvious since we use sequence and PPI network as input.

We also found that predicted genes have higher gene expression correlation with known annotated genes than random genes (Figure 4C), which suggesting the effectiveness of our predicted gene-phenotype association since we did not use gene expression data in training.

Known annotated genes and predicted genes associated with certain phenotype are supposed to be of the same biological functions. Here, we introduce the smallest shared biological process (SSBP) [33] to measure the functional similarity of a pair of proteins. SSBP of each pair of proteins was computed based on Gene Ontology by finding all the GO terms shared by them, and the GO term with the smallest protein count was identified. Protein pairs with smaller SSBP tend to share more specific GO terms. Although we did not use GO annotation for training, predicted genes still have statistically lower SSBP with known annotated genes compared with random genes (Figure 4D).

### 3.4. Discovery of Genes Associated with Abnormality of Mitochondrial Respiratory Chain

Mitochondrial oxidative phosphorylation is the major ATP-producing pathway, which supplies more than 95% of the total energy requirement in the cells. The mitochondrial respiratory chain (MRC) comprises ~92 nuclear and mitochondrial DNA-encoded protein subunits that are organized into five different multi-subunit respiratory complexes [34]. These complexes produce 90% of the ATP required for cell sustenance. Damage to the mitochondrial respiratory chain has been suggested to be an important factor in the pathogenesis of a range of psychiatric disorders [35,36]. Tissues with high energy demands, such as the brain, contain a large number of mitochondria, being therefore more susceptible to reduction in the aerobic metabolism [37].

**Figure 5:**
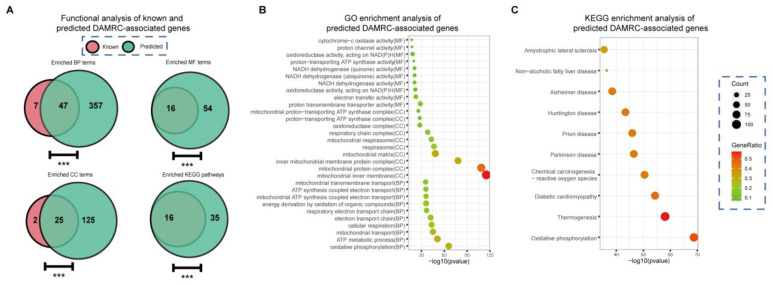
Functional analysis of predicted and known annotated genes with the phenotype of Decreased activity of mitochondrial respiratory chain. (**A**) Overlap between predicted and known annotated “Decreased activity of mitochondrial respiratory chain”-associated genes with respect to enriched GO terms and KEGG pathways. A relatively large shared GO enrichment terms and KEGG enrichment pathways was found. (*** *p*-value < 0.001, hypergeometric test). Predicted “Decreased activity of mitochondrial respiratory chain”-associated genes are enriched in mitochondrial related GO terms (**B**) and Mitochondrial related diseases such as Alzheimer’s disease, Parkinson’s disease, etc. (**C**).

We predicted 995 candidate DAMRC (HP:0008972)-associated genes using GraphPheno. In a quantitative functional analysis of these genes, we considered the enrichment of GO terms and KEGG pathways through a hyper-geometric test. We found that a relatively larger shared enriched GO terms and KEGG pathways appeared in the known annotated and predicted genes (Figure 5A). In particular, we found predicted DAMRC (HP:0008972)-associated genes were actively involved in mitochondrial inner membrane, mitochondrial protein complex, respiratory electron transport chain, etc. (Figure 5B). Pathway analysis using Reactome [38] shows that these genes are actively involved in oxidative phosphorylation, thermogenesis, and several neurodegenerative diseases (NDs) such as Parkinson’s disease [36], Huntington’s disease [35] and Alzheimer’s disease [39] (Figure 5C).

**Figure 6:**
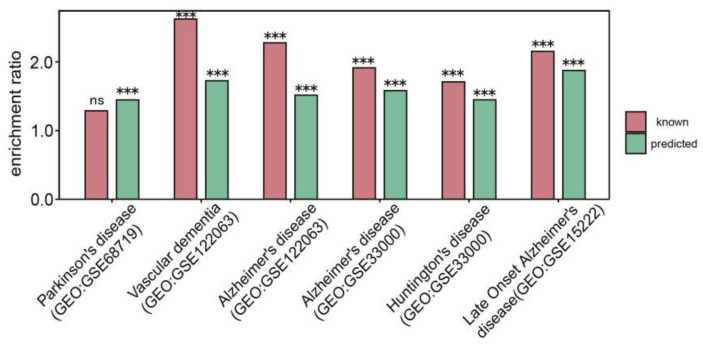
Both predicted and known annotated “Decreased activity of mitochondrial respiratory chain”-associated genes tend to be significantly down regulated in multiple neurodegenerative diseases. Enrichment ratio was calculated as the GeneRatio divided by Background Ratio. GeneRatio refers to the number of predicted or known annotated “Decreased activity of mitochondrial respiratory chain”-associated genes which are significantly down regulated in the GEO dataset divided by the total number of predicted or known annotated “Decreased activity of mitochondrial respiratory chain”-associated genes. Background Ratio refers to the number of significantly down regulated genes in the GEO dataset divided by the total number of genes identified in the GEO dataset. (ns: *p*-value > 0.05, *** *p*-value < 0.001, hypergeometric test).

Several studies demonstrate that there are reduced expression and impaired activity of respiratory chain Complexes in NDs [40]. We explored the gene expression level of those predicted and known DAMRC-related genes in multiple mitochondrial diseases-related expression data sets from GEO (GSE68719, GSE122063, GSE15222). As a result, we found that both predicted and known genes associated with DAMRC were enriched in significantly down-regulated genes in these datasets (Figure 6), indicating our predicted genes might be involved in these pathological process and act as potential biomarkers. R package “limma” (Linear Models for Microarray Data) [41] was used to identify significantly down regulated genes.

**Figure 7:**
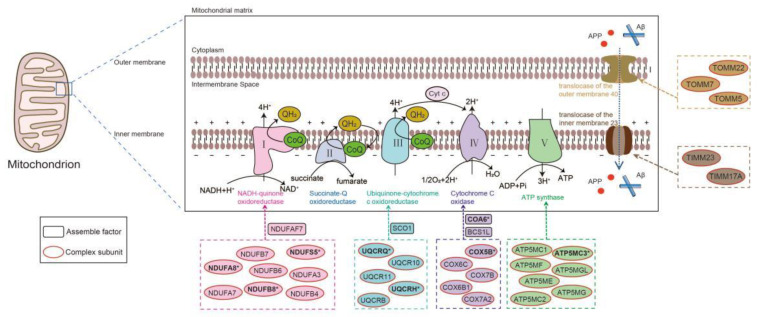
Biological insight into the predicted “Decreased activity of mitochondrial respiratory chain”-associated genes. The oxidative phosphorylation (OXPHOS) system is embedded in the lipid bilayer of the inner mitochondrial membrane (IMM) and is composed of five protein enzyme complexes and two mobile electron carriers namely ubiquinone (CoQ) and cytochrome c (Cyt C). Translocator of the outer and inner mitochondrial membrane (TOM and TIM, respectively) were also shown. Predicted “Decreased activity of mitochondrial respiratory chain”-associated genes were presented in dotted boxes, in which circle and rectangle denote genes which function as the subunits and assemble factors of mitochondrial respiratory chain complex I–V, TOM40 complex and TIM23 complex, respectively. Predicted “Decreased activity of mitochondrial respiratory chain”-related genes validated by recent papers or the newest version of HPO database were marked with asterisks.

Further on, we manually examined the top 150 prioritized DAMRC-associated genes. Among these 150 genes, 67 genes were associated with the assembly of mitochondrial respiratory chain complex I–V (53 genes were subunits of mitochondrial respiratory chain complex, 14 genes were assembly factors of mitochondrial respiratory chain complex and coenzyme Q), 5 predicted genes were subunits of translocase of the outer mitochondrial membrane (TOM) 40 and translocase of the inner mitochondrial membrane (TIM) 23 (Figure 7). These results are of biological significance considering that the predictions did not utilize any function and localization information about mitochondrial respiratory chain.

In more detail, *NDUFA8* (Rank:1) and *COA6* (Rank:15) were annotated to HP:0011923 (Decreased activity of mitochondrial complex I) and HP:0008347 (Decreased activity of mitochondrial complex IV), respectively, which are sub classes of HP:0008972 in the newest version of HPO database (December 2021).

Our predictions about 2 DAMRC-associated genes were validated by recent papers. An article published in November 2021 reported that *UQCRH* (Rank:8)-deficient mouse model shows impaired CIII activity [42]. *ATP5MC3* (Rank:24) encodes a structural complex V (CV) subunit, and heterozygous *ATP5MC3* variants were reported to reduce mitochondrial complex V activity in a paper published in October 2021 [43].

Moreover, GraphPheno has successfully identified 4 DAMRC-associated genes (*UQCRQ*, Rank: 6 [44]; *NDUFA5*, Rank: 10 [45]; *NDUFB8*, Rank: 13 [46]; *COX5B*, Rank: 16 [47]) which were omitted by HPO database. These findings suggest that our model is of certain prediction power for novel gene-phenotype associations discovery (Figure 7 and Supplementary Table S4).

GraphPheno also has the ability to find potential drug targets. For example, the predicted *PMPCB* (Rank:46, a subunit of mitochondrial-processing peptidase) is a member of the family of mitoproteases, which can modulate several biological activities necessary for proper mitochondrial functioning, such as apoptosis. *PMPCB* has been reported to be associated with the chemoresistance of HCC to sorafenib. Silencing of *PMPCB* can increase HCC tumor cell susceptibility to sorafenib therapy and is a potential drug target [48].

## 4. Discussion

Determining the HPO annotations of human genes can promote disease gene identification and prioritization and hence assist in clinical diagnostics. However, the current HPO annotations are still incomplete. To address this challenge, we developed a novel computational strategy called GraphPheno to formalize gene features via protein homology and protein interactions for automatic HPO annotation prediction. GraphPheno exhibits superior efficacy over routine strategies.

Case studies on mitochondrial related diseases show that GraphPheno has the ability to find novel gene-phenotype associations and potential drug targets. In fact, it is easy to extend GraphPheno to other diseases. For example, among the top 80 prioritized Hypercholesterolemia (HP:0003124)-associated genes (only 18 of them are membrane proteins) predicted by GraphPheno, the knockdown of *SOAT1* (Sterol O-acyltransferase 1, located in endoplasmic reticulum) (Rank:65) was reported to alter the distribution of cellular cholesterol, and effectively suppresses the proliferation and migration of hepatocellular carcinoma [49]. Furthermore, avasimibe, an inhibitor of *SOAT1*, was reported to markedly reduce the size of tumours that had high levels of *SOAT1* expression. Besides *SOAT1*, several proteins related to cholesterol homeostasis including *CYP7A1* (Rank:16, Cytochrome P450 7A1, located in endoplasmic reticulum), *HMGCR* (Rank:17, 3-hydroxy-3-methylglutaryl-coenzyme A reductase, located in endoplasmic reticulum) and *SREBF2* (Rank:45, Sterol regulatory element-binding protein 2, located in cytoplasm and endoplasmic reticulum) are reported to be significantly up-regulated in the HCC tumour tissue, suggesting these genes might be potential targets for HCC drugs [49] (Supplementary Table S5). In order to facilitate researchers to explore many other diseases/phenotypes, a comprehensive predicted gene-phenotype association dataset involving 4369 diseases/phenotypes was provided to facilitate the study of diseases/phenotypes (Supplementary Table S6). This dataset could be a valuable resource for biologists to find candidate genes for diseases/phenotypes research.

The sequence homology of proteins is very important for prediction. In GraphPheno, genes with similar sequences tend to group in the same functional category in the prediction output. For example, among the predicted phenotype terms for *CYP3A4* and *CYP3A5* (which are members of cytochrome P450 enzyme system (CYP) family 3 subfamily A), there are 4 overlap phenotypes: HP:0001939 (Abnormality of metabolism/homeostasis), HP:0011849 (Abnormal bone ossification), HP:0004349 (Reduced bone mineral density) and HP:0000818 (Abnormality of the endocrine system). Meanwhile, if we randomly selected two genes and count the number of overlap phenotypes, we found that the average number of the overlap phenotypes is significantly lower than that of *CYP3A4* and *CYP3A5* (0.5 vs. 4, empirical *p*-value < 0.01), which means GraphPheno can describe the commonalities and differences between protein sequences and lead to the grouping of paralogous genes with similar sequences in the same functional category in the prediction output. It should be pointed out that the baseline ‘Sequence’ method without integration of PPI network information has an average AUC of 0.497, which is worse than random, meaning it is difficult to learn vectors that can distinguish functional differences between proteins based on primary structures only. However, by integration of protein sequences and PPI network information, GraphPheno can obtain good predicting performance.

HPOLabeler can make use of wide variety of evidences from Gene Ontology, protein domains, trigram frequency and HPO term frequency to predict protein-HPO associations. It is the first method which has outperformed a simple Naive approach in the second Critical Assessment of Functional Annotation, 2013–2014 (CAFA2). In performance comparison under the benchmark of the CAFA2 [4], HPOLabeler has slightly higher *F*-max than GraphPheno (0.396 vs. 0.383, *p*-value = 1.92 × 10^−4^, U-test). It should be pointed out that GraphPheno only uses protein primary sequence and protein interaction data as input without any other prior knowledge. As announced by authors of HPOLabeler, temporal validation has low prediction performance, which might be caused by incomplete annotation of new proteins [13]. For those proteins without rich annotations, GraphPheno can be a good complement to HPOLabeler. We calculated the *F*-max of two methods (HPOLabeler and GraphPheno) against one independent test dataset of newly annotated 70 genes (HPO annotations added between 9 March 2018 and 21 December 2018) which have less than 10 GO annotations. The results show that GraphPheno was of significantly higher *F*-max than HPOLabeler (0.153 vs. 0.054, *p*-value = 2.22 × 10^−16^, Student’s *t*-test).

In general, we believe GraphPheno will contribute to novel gene-phenotype association discovery and be a valuable resource for disease/phenotypes research.

## Data Availability

All relevant data and codes are available at https://github.com/herry0310/graphpheno, accessed on 7 March 2022.

## Supplementary Materials

The following supporting information can be downloaded at: https://www.mdpi.com/article/10.3390/cells11162485/s1, Supplementary Table S1: Gold standard dataset; Supplementary Table S2: Comparison dataset; Supplementary Table S3: Relationship between amino acids and CT classes; Supplementary Table S4: Top 150 prioritized HP:0008972 (Decreased mitochondrial respiratory chain activity)-associated genes. Supplementary Table S5: Top 80 prioritized HP:0003146 (Hypocholesterolemia)-associated genes; Supplementary Table S6: Predicted gene-phenotype association dataset; Supplementary Figure S1: Statistics on groups of HPO terms.
