# Supplementary material for: Integration of Human Protein Sequence and Protein-Protein Interaction Data by Graph Autoencoder to Identify Novel Protein-Abnormal Phenotype Associations
Source: Cells. 2022 Aug 10;11(16):2485. doi: 10.3390/cells11162485 (PMC9406402; doi:10.3390/cells11162485)
Supplement: Supplementary file 1 [file cells-11-02485-s001.zip › Supplementary Figure S1.pdf]

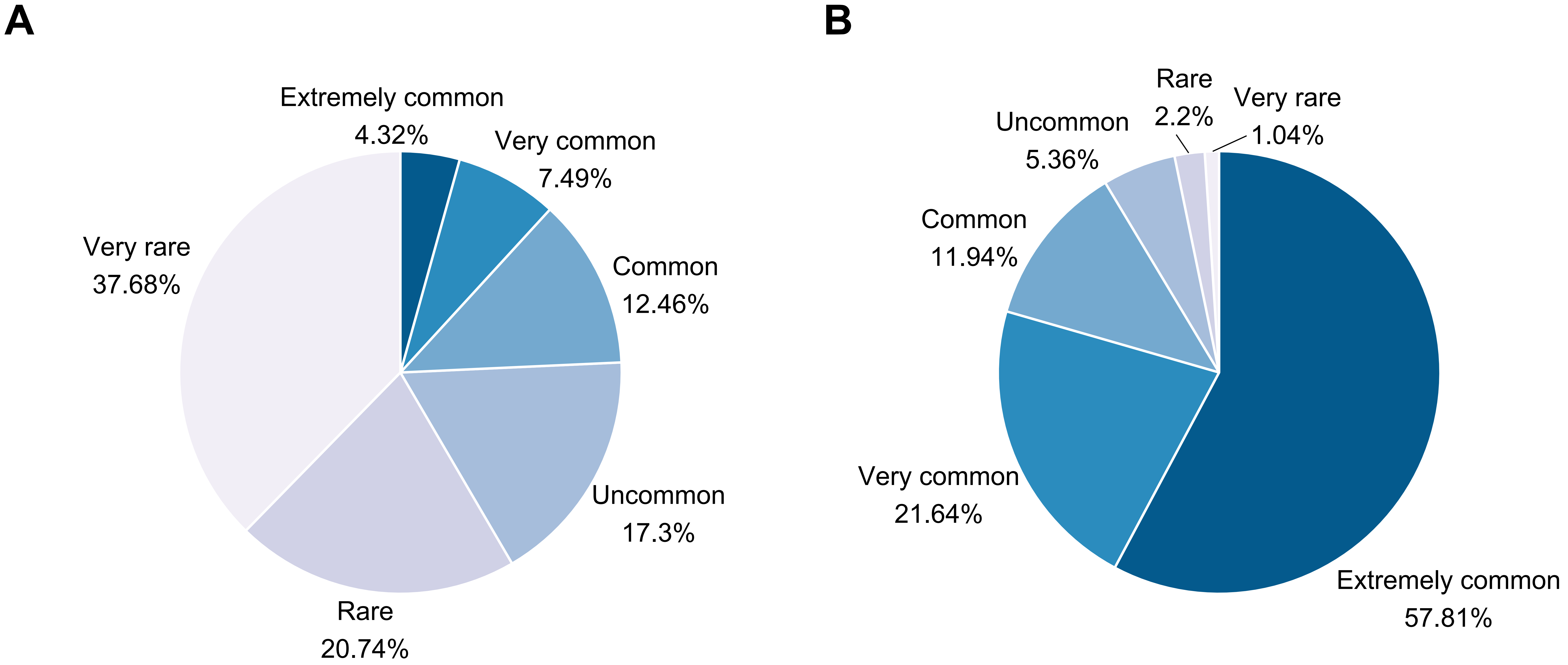

Supplementary Figure S1. Statistics on groups of HPO terms.
(A) The proportion of HPO terms in each group. (B) The proportion of annotated genes related to the terms in each group.
